# Performance Degradation Modeling and Its Prediction Algorithm of an IGBT Gate Oxide Layer Based on a CNN-LSTM Network

**DOI:** 10.3390/mi14050959

**Published:** 2023-04-28

**Authors:** Xin Wang, Zhenwei Zhou, Shilie He, Junbin Liu, Wei Cui

**Affiliations:** 1 School of Automation and Engineering, South China University of Technology, Guangzhou 510641, China; 2 China Electronic Product Reliability and Environmental Testing Research Institute, Guangzhou 511370, China; 3 Key Laboratory of Science and Technology on Reliability Physics and Application of Electronic Component, Guangzhou 511370, China

**Keywords:** IGBT, gate oxide layer degradation, feature fusion, performance prediction, CNN-LSTM network

## Abstract

The problem of health status prediction of insulated-gate bipolar transistors (IGBTs) has gained significant attention in the field of health management of power electronic equipment. The performance degradation of the IGBT gate oxide layer is one of the most important failure modes. In view of failure mechanism analysis and the easy implementation of monitoring circuits, this paper selects the gate leakage current of an IGBT as the precursor parameter of gate oxide degradation, and uses time domain characteristic analysis, gray correlation degree, Mahalanobis distance, Kalman filter, and other methods to carry out feature selection and fusion. Finally, it obtains a health indicator, characterizing the degradation of IGBT gate oxide. The degradation prediction model of the IGBT gate oxide layer is constructed by the Convolutional Neural Network and Long Short-Term Memory (CNN-LSTM) Network, which show the highest fitting accuracy compared with Long Short-Term Memory (LSTM), Convolutional Neural Network (CNN), Support Vector Regression (SVR), Gaussian Process Regression (GPR), and CNN-LSTM models in our experiment. The extraction of the health indicator and the construction and verification of the degradation prediction model are carried out on the dataset released by the NASA-Ames Laboratory, and the average absolute error of performance degradation prediction is as low as 0.0216. These results show the feasibility of the gate leakage current as a precursor parameter of IGBT gate oxide layer failure, as well as the accuracy and reliability of the CNN-LSTM prediction model.

## 1. Introduction

Insulated-gate bipolar transistors (IGBT) are a key power semiconductor component of power electronic converters. IGBTs have the advantages of high input impedance, high switching speed and low switching loss of semiconductor field effect transistor, and high current density, low saturation voltage drop, and strong current handling ability of bipolar transistors. For these reasons, IGBTs have been widely used in rail transit, new energy, communications, and other fields in recent years [1,2,3]. Due to the rapid development of power semiconductor manufacturing technology, the volume of IGBT modules is shrinking, but the external loading condition they need to bear is becoming much heavier, leading to a high failure rate. The aging failure of power devices will inevitably affect the normal operation of the entire system, and even cause huge economic losses in serious cases. Therefore, it is necessary to establish a reliable IGBT fault prediction system, predict the degradation trend of IGBTs throughout their life cycle, and assist the system in obtaining fault information in advance.

The main methods for IGBT failure prediction are divided into reliability-based life prediction methods and data-driven methods based on failure precursor parameters [4,5]. Reliability-based life prediction methods [6,7,8] include physical analytical models and statistical models. The physical analytical models are built on the failure mechanism of IGBTs, which predict stress, damage and reliability through finite element analysis or experiment. Based on the statistical method of data, the statistical models are used to fit the relationship between temperature average, temperature fluctuation value, and the number of failures. An electro-thermal stress analysis model for IGBT bonding line failure is established in [6]. For failure modes such as bonding wires and solder layers, a new aging analysis method is proposed based on Miner linear failure accumulation theory [7]. An accelerated life test scheme is given in [8] and used to evaluate the life of IGBTs under operating conditions. The reliability-based life prediction methods need the accurate failure mechanism model of the device, but it is difficult to obtain the parameters of the mechanism model and to predict the IGBT life in real time.

With the rapid development of artificial intelligence algorithms, many methods such as neural networks [9,10,11] and support vector machines [12] are used to build the prediction methods. The data-driven method based on fault precursor parameters [13,14,15,16,17,18,19,20] is built on parameters that can characterize the aging of IGBTs. This method determines the current operation status and predicts the future degradation of fault precursor parameters based on the collected data under historical operating conditions. The current research mainly extracts characteristic parameters from collector-emitter voltage data and predicts the degradation trend of IGBTs, which makes it possible to generate more accurate predictions through the historical data collected by the sensor without mastering the failure mechanism inside the IGBT. At present, the research on IGBT health evaluation and prediction focuses on the failure modes such as the latch-up effect and bonding line failure of IGBT chips. A series of modeling analyses are mainly carried out with respect to collector-emitter voltage. Ge et al. used the peak-to-peak of transient collector-emitter voltage as an indicator of IGBT performance degradation and published the DeepAR prediction model to predict the remaining lifetime of IGBT in [13]. Lu and Christou also extracted the performance degradation indicator from the collector-emitter voltage, and used the Long Short-Term Memory (LSTM) prediction model to predict the performance degradation trend of IGBT, in which the average absolute error can be predicted as low as 0.0322 [14]. Zhang [15,16] et al. extracted parameters such as saturation voltage drop and tailing current for fusion, introduced a comprehensive health indicator, and obtained IGBT health status assessment results. Liu et al. introduced a CNN1D-LSTM hybrid model to construct the IGBT life prediction model with respect to collector emitter voltage, which characterizes IGBT failure modes of weld layer failure and latch failure in [17]. Apart from all kinds of neural network algorithms widely used at present, the simple regression models also show potential in IGBT residual life prediction experiment. Ali [18] and Ismail [19] both introduced a Gaussian Process Regression (GPR) model to predict IGBT failure modes, and the experimental results have shown that the proposed prognostic approach is a powerful method. Further results about the data-driven prediction method can be found in [20,21,22].

In rail transit, new energy, communications and other fields, grid oxide degradation is one of the main failure mechanisms of IGBTs [23,24]. This failure mechanism can be characterized by collector-emitter voltage parameters as well as gate leakage current. The degradation of the IGBT gate oxide layer results in a small amplitude of gate leakage current change; therefore, the extraction of weak degradation features and modeling of degradation law need exquisite modeling skills. Since the acquisition of IGBT collector-emitter voltage involves a high voltage test in engineering practice, the volume of the monitoring module may be large. Accordingly, it is difficult to collect collector-emitter voltage in the limited space of high-speed train, communication and other equipment systems. In comparison, the monitoring module needed to collect gate leakage current is smaller, so it is more feasible to monitor and predict the degradation trend of gate leakage current (I_G_) caused by the degradation of gate oxide.

Therefore, the gate leakage current (I_G_) is selected as the precursor parameter of IGBT gate oxide degradation for feature extraction and degradation trend modeling in this paper. Firstly, I_G_ is analyzed in time domain, and combined with grey correlation degree, Mahalanobis distance and the Kalman filtering method, and multi-dimensional characteristics are fused into a health indicator, which can characterize the degradation degree of IGBT gate oxide. Secondly, this paper introduces a Convolutional Neural Network and Long Short-Term Memory (CNN-LSTM) network to obtain the real-time prediction of performance degradation. Finally, this paper uses the dataset published by NASA Ames Laboratory to verify the effectiveness of the CNN-LSTM network, and discusses the prediction error using different prediction models.

## 2. Analysis of IGBT Failure Modes and Their Precursor Parameters

The degradation of the health state of IGBT is a process of gradual evolution under the action of external stress, and the degradation of each precursor parameter of IGBT is monitored in real time by condition monitoring technology, and the potential failure can be detected in advance when the health state of IGBT is approaching failure. This section gives a brief overview on the failure modes of IGBT and their common precursor parameters.

### 2.1. Failure Mode Analysis

The failure mode of IGBT is mainly divided into two categories such as chip internal failure and device package failure. The chip internal failure modes include overstress failure, latch-up effect and gate failure, mainly caused by the electrical overstress and charge effect existing inside the chip. These failure modes are manifested in the form of secondary device breakdown, latch-up failure, gate oxide layer breakdown, etc.

The package-related failure is mainly caused by the different coefficients of thermal expansion of the metal materials in the IGBT package, and the structure of the connecting part is constantly affected under the action of thermal stress, which eventually leads to the failure phenomena, such as bond wire shedding, solder layer aging and aluminum metal reconstruction.

### 2.2. Selection of Precursor Parameters

The studies [6,7,8,9,10,11,12,13,14,15,16,17,18,19,20,21,22,23,24] show that the IGBT failure precursor parameters can directly or indirectly reflect the health state of the IGBT, mainly including collector-emitter voltage (V_CE_), gate-emitter threshold voltage (V_GEth_), collector shutdown current (I_C_), gate leakage current (I_G_), junction temperature and thermal resistance, etc.

V_CE_ can be used as a precursor parameter for both failure modes of weld layer failure and latch failure. The latch-up effect caused by chip bonding tends to cause V_CE_ to decrease, while bond wire detachment failure tends to increase V_CE_ values. Due to the decline in V_CE_ caused by chip bonding and the loss of bonding wires, the increase in V_CE_ value has a high impact, and it is generally believed that the V_CE_ value increases as IGBT ages. The V_CE_ is the most commonly used health monitoring parameter due to its easy detection of V_CE_ through sensors under laboratory conditions [13,14,15,16,17,18,19,20]. The part of the shutdown current I_C_ in the slow falling phase of the IGBT is called the tail current, and the shutdown current duration can be used as a performance parameter to characterize the IGBT latch-up effect, so the tailing current can also be used as an electrical parameter to characterize the IGBT latch-up effect.

The gate-emitter threshold voltage V_GEth_ and gate leakage current I_G_ can be used to characterize the degradation of the gate oxide layer of IGBT devices [23,24]. As the device’s aging process changes, the degradation of IGBT performance affects the internal structure of the device’s gate oxide layer, and the change in the oxide layer will cause the gate capacitance parameters to also change. Although V_GEth_ can be monitored by certain techniques under laboratory conditions, it is currently difficult to monitor the gate emitter voltage using sensors in practical application sites. In contrast, the monitoring circuit of the gate leakage current I_G_ is small, and this parameter can be used as a precursor parameter for the performance degradation of the gate oxide layer of IGBT devices in engineering.

Junction temperature and thermal resistance are mainly used to characterize package-related failure modes such as IGBT solder layer degradation [6,7,8]. An easy way to measure junction temperature is to place a temperature sensor close to the module junction, capable of measuring junction temperature online without interrupting the normal operation of the IGBT. However, this method is invasive, and the accuracy of the measurement is affected by the sensor position. If the temperature and heat generation are known, in order to easily measure the actual solder damage parameters within the IGBT module, an accurate electro-thermal model needs to be established to calculate the thermal resistance and power lost.

In summary, the precursor parameters of IGBT gate oxide degradation failure are gate-emitter threshold voltage and gate leakage current. Because the online detection required to collect gate leakage current is easy to achieve in engineering, this paper takes gate leakage current as the precursor parameter to carry out IGBT gate oxide degradation modeling and prediction research, which provides a method model for online detection, feature extraction and health state prediction of IGBT gate oxide performance degradation.

### 2.3. Analysis of Performance Degradation Mechanism and Its Precursor Parameter of Gate Oxide Layer

The change in the gate leakage current characteristics of IGBT is mainly caused by the degradation of the performance of the gate oxide layer of the device, the inherent pinhole defect of the gate silica film insulation layer, or late fatigue aging. This will cause the IGBT to undergo gate Al-SiO_2_ interface ion diffusion and increase Si-SiO_2_ interface charge density, among other changes. This is due to the presence of electron traps, hole traps, and neutral traps in the gate oxide layer, which, when the oxide tunnel has current through the trap, will capture carriers and accumulate positive or negative charge. The accumulated movable ion charge, interface trap charge, oxide trap charge, etc., will enhance the local electric field of the oxide layer, further increasing the leakage current, and, eventually, the gate oxide layer will breakdown, causing the gate control to fail. Therefore, gate leakage current I_G_ can be used as a precursor parameter for the degradation failure of the IGBT gate oxide layer.

For this reason, I_G_ is used as the precursor parameter of the performance degradation failure of the IGBT gate oxide layer. The performance degradation indicator of IGBT is obtained by time domain analysis, feature selection, fusion, and noise reduction, and the CNN-LSTM network is introduced to predict the performance degradation of IGBT, while the effectiveness of the prediction algorithm is verified by the open dataset.

## 3. Construction of Performance Degradation Indicator of IGBT Gate Oxide Layer

For the performance degradation of the IGBT gate oxide layer, the performance degradation indicator can visually characterize the aging degree of the gate oxide layer of IGBT, which is the input of the prediction model. When the model prediction result reaches the failure threshold, it means that the IGBT fails.

**Figure 1:**
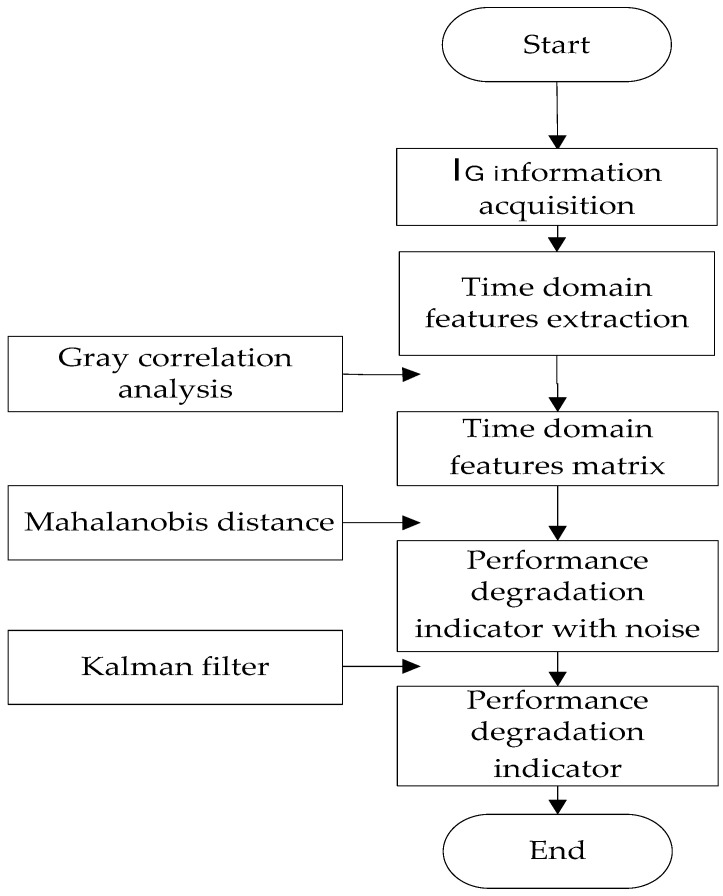
Construction of Performance Degradation Indicator of IGBT.

In this paper, the 13 time-domain features such as maximum, minimum, variance and skewness, etc., are extracted by time domain analysis of the I_G_, and, then, the IGBT performance degradation indicator MD is obtained through feature selection, fusion, and noise reduction. The construction of this performance degradation indicator is shown in Figure 1. To be more specific, this construction process is given in Section 3.1, Section 3.2 and Section 3.3, respectively.

### 3.1. Precursor Parameter I_G_ Information Acquisition

In this paper, the IGBT aging experimental data released by NASA were used, and the last 700 cycles of data at the end of the experiment were selected for experimental analysis for secondary acquisition, and the last 10 cycle data after IGBT failure were excluded. For IGBT, one turn-on and one turn-off action amounts to once cycle. In this paper, the gate leakage current I_G_ is used as a precursor parameter to the failure of the gate oxide layer, and when the IGBT is turned off, the small current flowing through the gate is I_G_.

**Figure 2:**
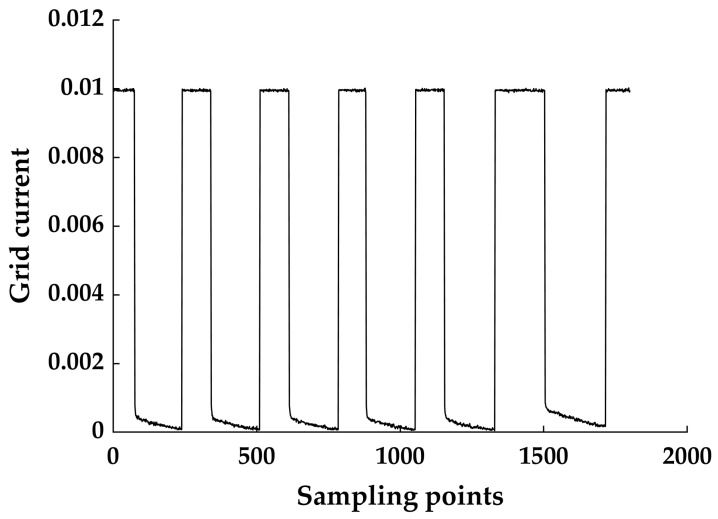
Dynamic curve of grid current.

Figure 2 shows part of the dynamic curve of the gate current, and it can be observed that the current gap between the on-time and the off-time is significant, so the threshold value can be set for collecting I_G_.

### 3.2. Gray Correlation Calculation of Time Domain Features

The time domain features of the 13 extracted time-domain features do not all contain the degradation information of the gate oxide layer of IGBT, and the development trends of different features have certain similarities, which is very suitable for studying the correlation within each other by gray correlation, so as to further extract the necessary features. The correlation calculation formula used in this paper is as follows:

(1) $${\gamma (X}_{0}{,X}_{i})=\frac{1}{n}\sum_{k=1}^{n}\xi_{i}{(x}_{0}{(k),x}_{i}\left(k)\right)$$

where is the difference between the sequence X_0_ and X_i_ at each point, and its calculation method is given in Equation (2), while ζ represents the resolution coefficient, and the value range is (0, 1).

(2) $$\xi_{i}{(x}_{0}{(k),x}_{i}(k))=\frac{\mathrm{minmin}\left|x_{0}{(k)-x}_{i}\left(k\right)\right|+\zeta \mathrm{maxmax}\left|x_{0}{(k)-x}_{i}\left(k\right)\right|}{\left|x_{0}{(k)-x}_{i}\left(k\right)\right|+\zeta \mathrm{maxmax}\left|x_{0}{(k)-x}_{i}\left(k\right)\right|}$$

**Figure 3:**
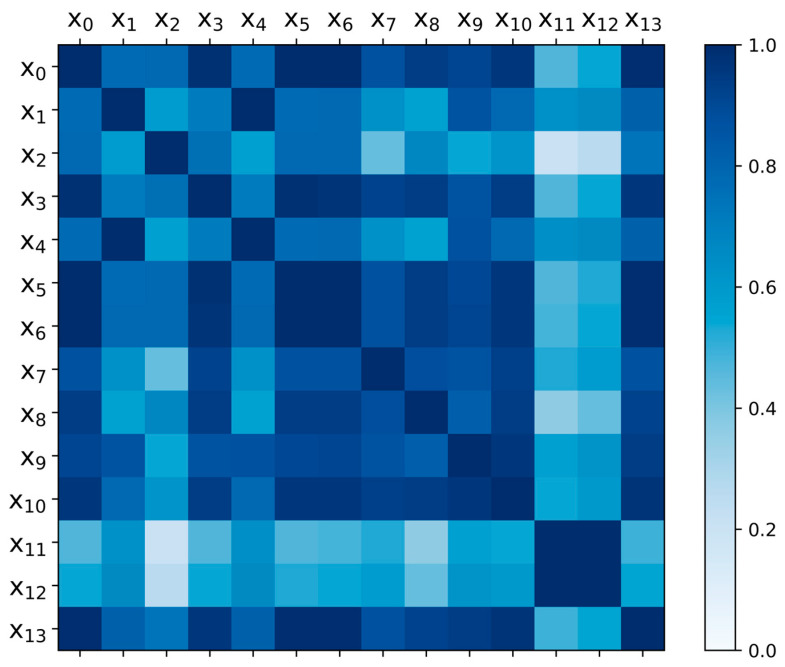
Thermal diagram of correlation f I_G_ time-domain feature.

Finally, the thermal diagram of the correlation degree of I_G_ characteristics in each time domain is obtained as shown in Figure 3.

The shade of color in Figure 3 indicates the degree of correlation between the two sequences, and it can be deduced that the correlation between different features is high or low, and the correlation between features and cycle periods is also different. The degradation of the gate oxide layer of IGBT is a time-sensitive process, so the features highly correlated with the cycle should be the available features with more degradation information. In order to avoid the relevance between different features affecting the results, the features with high correlation are further eliminated. Finally, the selected I_G_ time domain features include RMS root mean square, steepness and kurtosis.

### 3.3. Features Fusion and Nosie Reduction

In Section 3.2 of this paper, a well-performing time domain feature has been selected, but a unified indicator is required to characterize the performance degradation of IGBTs, and the acquired data contains a lot of noise and needs to be reduced. In this paper, the feature matrix is built based on Mahalanobis distance (MD), and the noise is reduced by the Kalman filter theory.

The larger the Mahalanobis distance (MD) is, the greater the deviation between sample sets, which is consistent with the degradation process of IGBT. There are sample set X and sample set Y, where the X is a row vector composed of n sample points, and the Y is m × n-dimensional sample set. The Mahalanobis distance between X and Y is calculated as shown in Formula (3).

(3) $$\mathrm{MD}=\sqrt{\left|\left(X-\overset{-}{Y}\right)\sum^{-1}{\left(X-\overset{-}{Y}\right)}^{T}\right|}$$

where $\overset{-}{Y}$ is the barycenter of Ym × n, and Σ^−1^ is covariance of Ym × n.

**Figure 4:**
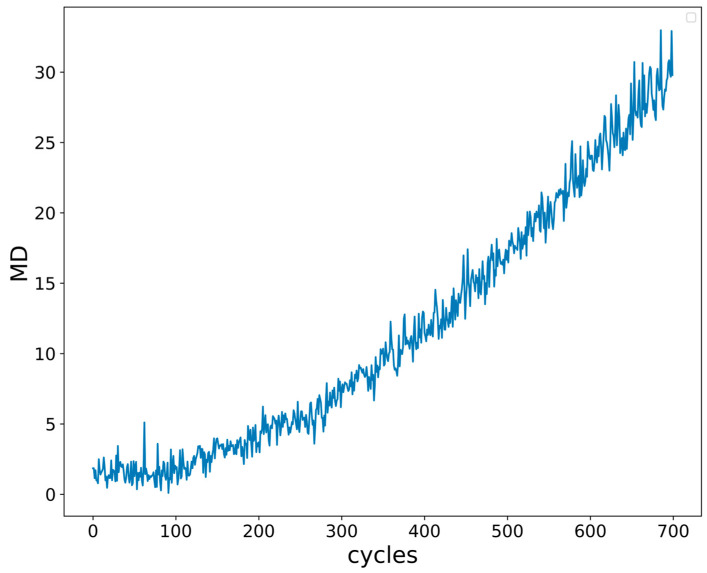
Mahalanobis distance value of I_G_.

Figure 4 shows that the ordinate is MD value of I_G_ and the abscissa is the IGBT working cycle number. With the increase in cycle, I_G_ gradually ages and MD also gradually increases, indicating the rationality of MD as an indicator of IGBT performance degradation.

Kalman filtering is a commonly used filtering algorithm to reduce signal noise in acquired data, which has higher accuracy and better interpretability in the field of signal processing. In this paper, the Kalman filter algorithm is further used to reduce the noise in MD value, forming into another denoised MD value. For convenience, it is also denoted as MD to prevent ambiguity.

## 4. Performance Degradation Prediction of Gate Oxide Layer Based on CNN-LSTM Network

In this paper, the CNN-LSTM network is used to construct an IGBT performance degradation prediction model, which ensures the accuracy of the prediction results through the powerful feature extraction capability of the Convolutional Neural Network (CNN) and the reliability of the Long Short-Term Memory (LSTM) model in the field of time series forecasting. The model input is a one-dimensional IGBT performance degradation indicator matrix, and the output is an IGBT real-time degradation indicator. There are two groups in the control experimental group, which use CNN, LSTM and Support Vector Regression (SVR) models, respectively.

### 4.1. CNN-LSTM Network

CNN convolves the local region of the input signal with the filter core through the convolutional layer, and then generates nonlinear features through the activation function [25]. For CNNs to process time series data, a one-dimensional convolutional network Con1d is typically used, with the following expression:

(4) $$x_{i}^{l}(j)=f(X^{l-1}(j)*k_{i}^{l}+b_{i}^{l})$$

where $k_{i}^{l}$ is the weight matrix of the *i*-th convolution kernel of the *l*-th layer, $X^{l-1}$ is the *j*-th local output of *l* − 1, $b_{i}^{l}$ is the bias, * is the convolution operation. Moreover, f is the activation function. This experiment uses the Relu function and its expression is as follows:

(5) $$f{(x)=x}^{+}=\max(0,x)$$

The long short-term memory network (LSTM) is often used to process time series data, and their structure consists of a forget gate, input gate, and output gate. Useless information is discarded through the forget gate with the following expression:

(6) $$f_{t}=\sigma (W_{f}[h_{t-1},x_{t}]+b_{f})$$

where $h_{t-1}$ is the output of the prior unit, $x_{t}$ is the input to the current cell, σ is the sigmoid activation function, and $W_{f},b_{f}$ are the weight matrix and the bias, respectively.

The role of the input gate is to decide to update the information, and the activation function uses the Relu function. The expression is as follows:

(7) $$\begin{array}{l} i_{t}=\sigma (W_{i}[h_{t-1},x_{t}]+b_{i})\\ C_{t}^{\prime }=\mathrm{Relu}(W_{C}[h_{t-1},x_{t}]+b_{C}) \end{array}$$

where $C_{t}^{\prime }$ is the unit state at the current moment, $W_{i},b_{i}$ are the weight matrix and bias of the input gate, respectively, and $W_{C},b_{C}$ are the weight matrix and bias for the current state, respectively.

The output gate then determines the current output information $o_{t}$ and obtains the output $h_{t}$ of the LSTM unit based on the updated cell state, i.e.,:

(8) $$\begin{array}{l} C_{t}=f_{t}^{}*C_{t-1}+i_{t}*C_{t}^{\prime }\\ o_{t}=\sigma (W_{o}[h_{t-1},x_{t}]+b_{o})\\ h_{t}=o_{t}*\mathrm{Relu}(C_{t}) \end{array}$$

where $W_{o},b_{o}$ are the weight matrix and bias of the output gate, respectively.

### 4.2. Training Procedure of Performance Degradation Predictor Based on CNN-LSTM Network

**Figure 5:**
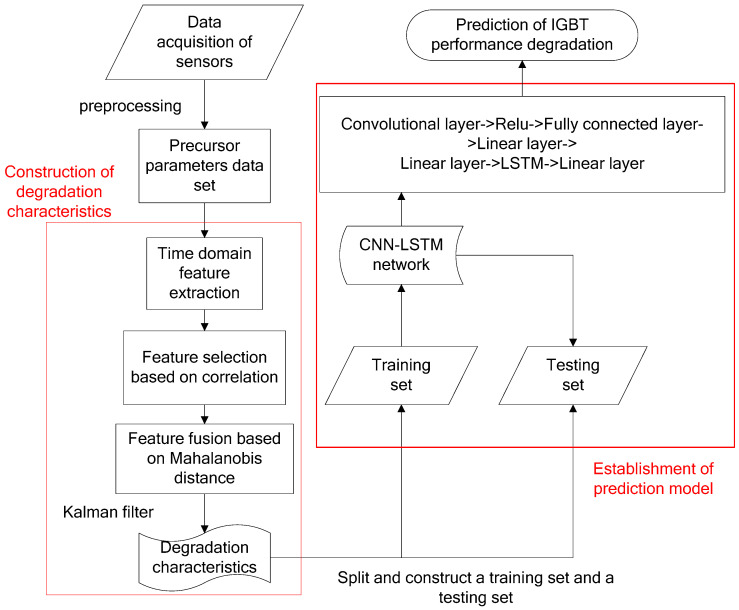
Flow chart of IGBT degradation prediction.

The flow of the IGBT gate oxide performance degradation prediction experiment and the CNN-LSTM network structure are shown in Figure 5.

The left part of Figure 5 is the feature engineering of IGBT gate aging data. During this process, about 400,000 sampling moments collected by the sensor are preprocessed. After feature engineering, 700 cycles of performance degradation indicators are obtained. Note that the performance degradation prediction of IGBT is highly correlated with historical state data, rather than the corresponding relationship between time and state. Therefore, a time window of 5 is set in this paper. Each time the performance degradation indicators of four cycles are fed into the prediction network, the prediction value of the next cycle will be given by the network. 

The training process of the predictor and the concrete structure of the CNN-LSTN network are shown in the right part of Figure 5. Performance degradation indicators of 700 cycles are split into training set and test set; the training set is used to train the predictor to fit it, and the test set is used to test the accuracy of the trainer.

In this paper, the root mean square error loss function (MSE loss function) is used to calculate the loss between the prediction value and the true value, and the adaptive moment estimation (Adam optimizer) is adopted for model optimization. The initial learning rate is set to 0.001, and the model can converge within 150 iterations. After the training, the model and corresponding parameters will be saved and used to predict the test set.

### 4.3. Prediction Experiment on the Performance Degradation of IGBT Gate Oxide Layer

In this paper, I_G_ is used as the precursor parameter of gate oxide degradation to construct the performance degradation indicator of IGBT gate oxide, and an IGBT performance degradation prediction model is built based on the CNN-LSTM network. The data of 500 cycles is trained first, and then the IGBT is predicted in real time within 200 cycles. In order to verify the effectiveness of the CNN-LSTM network, GPR, SVR, LSTM and CNN networks are used to construct IGBT performance degradation prediction models for controlled experiments.

**Figure 6:**
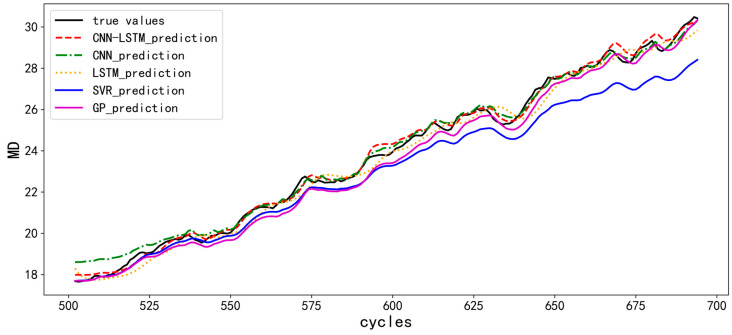
Forecast curves of CNN-LSTM, CNN, LSTM, SVR and GP models.

In Figure 6, the abscissa is the cycle period of the IGBT, and the ordinate is the performance degradation indicator of the IGBT, that is, MD. In the Figure 6, the SVR prediction curve and LSTM prediction curve deviated greatly from the actual value. The CNN prediction curve has a large prediction deviation in the first 50 cycles, and the fitting results later improve. The CNN-LSTM forecast curve performs very well throughout the whole forecast cycle. The accuracy of different models is quantitatively compared using mean absolute error (MAE) and the result is shown in Table 1.

In summary, the IGBT prediction model based on CNN-LSTM has the most accurate prediction results, and the MAE can reach as low as 0.0216.

## 5. Conclusions

In view of the degradation of IGBT gate oxide, this paper takes the gate leakage current as a failure precursor parameter, analyzes it in time domain, and obtains a performance degradation indicator (i.e., MD) that can characterize the IGBT gate oxide layer through feature selection, feature fusion, and noise reduction processing. In terms of the performance degradation prediction of the IGBT gate oxide layer, a prediction model based on the CNN-LSTM network is constructed, and the effectiveness of the model is verified by using NASA’s public dataset. The main contributions of this paper are as follows.

(1) Compared with previous studies [13,14,15,16,17,18,19,20] on prediction models of failure modes such as the weld layer failure, latch failure, and solder layer degradation, this paper focuses on the gate oxide layer performance degradation of IGBT and its corresponding failure precursor analysis, filling a void regarding the prediction of gate oxide layer performance degradation.
(2) In order to mine the small change in the gate leakage current, a series of gate leakage current analysis techniques, such as feature selection, feature fusion, noise reduction processing, Mahalanobis distance, and Kalman filter are used to extract the performance degradation indicator MD of the gate oxide layer, which gradually increases with the increase in the cycle, characterizing the gradual aging process of IGBT as a failure.
(3) The CNN-LSTM network is introduced to predict IGBT performance degradation prediction, which inherits the advantages of CNN and LSTM. The results show that the average absolute error in IGBT prediction experiments is 0.0216, which is lower than the prediction error of CNN, LSTM, and the SVR model.

In summary, this paper constructs an IGBT gate oxide performance degradation prediction model based on CNN-LSTM, selects gate leakage current as a precursor parameter, and obtains a unified indicator MD that can characterize IGBT aging. The final average absolute error of degradation prediction is as low as 0.0216, which indicates the feasibility of gate leakage current as a precursor parameter and the accuracy and reliability of the CNN-LSTM prediction model.

## Figures and Tables

**Table 1:** MAE of different prediction models.

Prediction Model	MAE
CNN-LSTM	0.0216
LSTM	0.0422
CNN	0.0312
SVR	0.7790
GPR	0.3949

## Data Availability

The data set used in this paper for supporting that our proposed framework is effective for aging prediction of IGBT grid oxide layer was provided by the NASA Prognostics Center of Excellence (PCoE). The data for download is available on https://data.nasa.gov/download/nk8v-ckry/application%2Fzip, accessed on 20 April 2023.
